# Platelet Indices in Patients With Gram-Negative and Gram-Positive Sepsis: A Retrospective Cross-Sectional Study

**DOI:** 10.7759/cureus.71601

**Published:** 2024-10-16

**Authors:** Josipa Tomic, Sanja Jakovac, Tanja Zovko, Ivona Ljevak, Sandra Karabatic, Marjana Mucic, Danijel Pravdic

**Affiliations:** 1 Department of Microbiology and Molecular Diagnostics, University Clinical Hospital Mostar, Mostar, BIH; 2 Department of Pulmonary Diseases and Tuberculosis, University Clinical Hospital Mostar, Mostar, BIH; 3 Clinic for Pulmonary Diseases, University Hospital Centre Zagreb, Zagreb, HRV; 4 Faculty of Health Studies, University Clinical Hospital Mostar, Mostar, BIH; 5 Clinic for Internal Diseases, University Clinical Hospital Mostar, Mostar, BIH

**Keywords:** gram-negative bacteria, gram-positive bacteria, platelet indices, platelets, procalcitonin, sepsis

## Abstract

Objectives: Different inflammatory processes and sepsis can significantly affect the number of platelets and platelet indices. Therefore, in this study, total platelet count (PLT), thrombocrit (Pct), platelet distribution width (PDW), mean platelet volume (MPV), and platelet-large cell ratio (P-LCR) were analyzed in patients with Gram-negative and Gram-positive bacterial sepsis and in sterile blood cultures.

Materials and methods: Inclusion criteria were an increased number of inflammatory parameters (elevated values ​​of leukocytes, C-reactive protein (CRP), procalcitonin (PCT), and positive blood culture. Exclusion criteria were patients who did not have elevated values ​​of inflammatory parameters and did not have a positive blood culture. Samples were collected from patients who had sepsis confirmed by blood cultures at the Department of Microbiology and Molecular Diagnostics at University Clinical Hospital Mostar in the period from 2019 to 2022. Three groups were analyzed, patients who had sterile blood cultures, patients with blood cultures with isolated Gram-positive bacteria, and patients with blood cultures with isolated Gram-negative bacteria. Specific infectious agents were identified for each group of patients. In addition to the above, PLT, Pct, MPV, PDW, P-LCR, PCT, CRP, the total number of leukocytes, and the number of neutrophil leukocytes were analyzed in each group.

Results: The values of PCT, CRP, and the number of neutrophile leukocytes were significantly higher in patients with Gram-negative sepsis as compared to Gram-positive sepsis and to control group. Patients with sepsis have decreased PLT and Ptc and increased values of MPV, PDW, and P-LCR. In sepsis caused by the Gram-negative bacteria, i.e., *Escherichia coli*, *Klebsiella pneumoniae*, and *Acinetobacter baumannii*, the values of the same parameters were more changed compared to sepsis caused by Gram-positive bacteria, i.e., *Streptococcus pneumoniae*, *Enterococcus *spp., and methicillin-resistant *Staphylococcus aureus *(MRSA). When comparing Gram-negative negative bacteria, PLT was lowest in sepsis caused by *Escherichia coli, *the PDW value was highest in sepsis caused by *Acinetobacter baumannii*, and MPV and P-LCR were the highest in sepsis caused by *Klebsiella pneumoniae*.

Conclusion: Our study showed that platelet indices are significantly changed in patients with sepsis. Patients with sepsis have decreased values of PLT and Pct and increased values of MPV, PDW, and P-LCR, indicating an increase in thrombocyte production. Moreover, the results were more prominent in sepsis caused by Gram-negative bacteria compared to sepsis caused by Gram-positive bacteria.

## Introduction

Sepsis is one of the biggest challenges with high mortality rates [1,2,3]. The European Society of Intensive Care Medicine and the Society of Critical Care Medicine define sepsis as a life-threatening organ dysfunction caused by a dysregulated host response to infection [4]. Globally, an estimated 48.9 million cases of sepsis occur annually, resulting in over 11 million deaths (19.7% of all deaths worldwide) [5]. One of the main challenges in managing sepsis is the absence of a "gold standard" in diagnosis. Diagnostic criteria such as fever, hypothermia, leucocytosis, leukopenia, and tachycardia are limited by specificity for sepsis [6,7,8]. Therefore, additional markers like platelet indices could be helpful in early diagnosis and therapeutic guidelines [8].

Platelets are blood particles without a nucleus that are formed in the bone marrow from parts of megakaryocytes and rapidly disintegrate, with a lifespan of three to five days [9]. Their number increases under the influence of inflammatory cytokines, whose activity is changed in sepsis, while the breakdown of platelets mostly takes place in the spleen, a key immune organ [10]. Therefore, various inflammatory processes in sepsis can significantly affect the number of platelets and platelet indices [11]. In previous studies, it has been shown that sepsis can affect the platelet indices [11,12], but the effect of different Gram-positive and Gram-negative bacteria on platelets was not studied. The mentioned parameters were studied in earlier studies in various pathological conditions such as pancreatitis, acute appendicitis, and infective endocarditis. The role of these parameters in sepsis is still insufficiently investigated [11]. The platelet-large cell ratio (P-LCR) serves as a proxy indicator for platelet volume, identifying the proportion of the largest platelets. An increase in P-LCR usually indicates an increase in new platelets of a larger size. Platelet distribution width (PDW) increases with a decrease in the number of platelets, and similar changes are observed in the mean platelet volume (MPV) during acute severe infections. Plateletcrit (Pct) is directly related to the number and size of platelets [11,12]. Thrombocytopenia indicates a poor prognosis associated with a higher risk of bleeding, organ dysfunction, and a worse outcome of sepsis treatment [13,14,15]. Septic shock is determined by changes in coagulation and platelet function. This patient’s condition is manifested by prolongation of the activated partial thromboplastin and prothrombin time and a decrease in the number of platelets [16].

Procalcitonin (PCT) is a sensitive clinical marker for the early detection of bacterial sepsis [16,17,18]. PCT levels >2 ng/mL suggest sepsis, while levels >10 ng/mL indicate septic shock [19]. Increased PCT levels have been evident in patients with Gram-negative bacterial sepsis [20,21,22,23]. Therefore, we used PCT levels to confirm septic response in patients with positive blood cultures.

Infections caused by Gram-negative bacteria can represent a significant challenge because of increasing antibiotic resistance and limitations due to treatment options [24]. Prompt sepsis diagnosis and prevention of complications are crucial for positive treatment outcomes. Gram-negative bacteria such as* Escherichia coli*, *Acinetobacter baumannii*, *Klebsiella pneumoniae*, and *Pseudomonas* *aeruginosa* are common causes of sepsis, particularly in hospitalized patients with postsurgical or underlying immunocompromised conditions [25].

The main objective of our study was to determine the total platelet count (PLT), Pct, PDW, MPV, and P-LCR in patients with Gram-negative bacterial sepsis, Gram-positive bacterial sepsis, and sterile blood cultures. Additional to these parameters values ​​of PCT, C-reactive protein (CRP), leukocytes, and neutrophil leukocytes were monitored. Moreover, the effect of infection caused by *Escherichia coli*, *Acinetobacter baumannii*, *Klebsiella pneumoniae*, *Streptococcus pneumoniae*, *Enterococcus spp.*, and methicillin-resistant *Staphylococcus aureus* on thrombocyte indices were analyzed.

## Materials and methods

Study design and study population

In this retrospective cross-sectional study, in patients with a diagnosis of sepsis (positive blood samples including cultures with Gram-positive and Gram-negative bacteria), the following parameters were analyzed: PLT, Pct, PDW, MPV, P-LCR, procalcitonin levels, CRP, total number leukocytes, and number of neutrophil leukocytes.

Inclusion criteria were positive blood culture and increased values of inflammatory parameters (elevated values ​​of leukocytes, CRP, and PCT). Exclusion criteria were patients who did not have elevated values ​​of inflammatory parameters and did not have positive blood cultures. Sepsis was defined by the sepsis-3 diagnostic criteria [26]. The data were collected at the University Clinical Hospital in Mostar from 2019 to 2022. 

Data collection

The samples from 120 patients were analyzed based on the statistician's assessment: 50 with Gram-positive bacteria, 50 with Gram-negative bacteria, and 20 patients with negative blood cultures.

Data were collected at the following departments of the University Clinical Hospital in Mostar: Intensive Care Unit, Department of Infectious Diseases, and Department of Neurosurgery. Laboratory values were analyzed at the Department of Laboratory Diagnostics of the University Clinical Hospital in Mostar. Data with positive blood cultures were collected at the Department of Microbiology and Molecular Diagnostics at the University Clinical Hospital in Mostar. The primary outcomes of the study were PLT, Pct, PDW, MPV, and P-LCR. The secondary outcome of the study was neutrophil count.

The platelet number and PLT were determined from venous blood. Two methods were applied to monitor these parameters: flow cytometry and hydrodynamic focusing method. MPV, PDW, and P-LCR measurements were conducted using hydrodynamic focusing techniques (Sysmex XN1000, Sysmex, Japan). The results were available within 24 hours.

Procalcitonin was measured from serum samples, using the same type of sample in order to monitor this marker. The measurement unit for the sample is ng/mL (sensitivity values < 0.5 ng/mL). These parameters were processed by Architect 8200i, Abbot, USA, within eight hours after laboratory testing.

CRP was processed from serum samples, applying turbidimetry (DxC 700 AU, Beckman Coulter, USA). The results were available within four hours after laboratory admission, with a minimum required sample volume of 0.5 mg/L. The reference values are <6 mg/L.

Leukocyte and neutrophil values were processed from venous blood by applying flow cytometry with a semiconductor laser (Sysmex XN1000, Sysmex, Japan). The results were available within 24 hours.

To detect blood infection, a minimum of two bottles of blood were needed, a volume of 8-10 ml, one vial for aerobic cultivation and one for anaerobic cultivation. The bottle sample was placed in the Bact/Alert 3D blood culture system (BioMerieux, Marcy-l'Étoile, France). To plant the growth of Gram-positive bacteria, 5% blood agar is used. In order to observe the growth of Gram-negative bacteria, MacConkey agar and Columbia agar for anaerobic bacteria were used. SS agar is used for *Salmonella *and *Shigella* bacteria detection. Sabourand agar is a selective medium designed for the growth of fungi while inhibiting the growth of bacteria. The blood cultures were incubated aerobically at 37 °C and observed for five to seven days.

This clinical study was approved by the Ethics Committee of the University Clinical Hospital Mostar where the study was conducted (no. 7275/24). Our study was performed following the ethical standards of the Helsinki Declaration of 1975, as revised in 2000.

Statistical analysis

Using one-way analysis of variance (ANOVA), differences in the average values of parameters (values ​​of platelets, mean platelet volume, distribution of platelets by volume, and elevated values ​​of procalcitonin, and other laboratory analysis values ​​necessary for research were used: C-reactive protein, leukocytes, and neutrophil leukocytes) were tested. Pearson correlation was used to test the correlation between the data. Diagnostic performance for sepsis was tested using the area under the curve (AUC) of receiver operating characteristic (ROC) curves, sensitivity, and specificity. The sample size of the groups was calculated for the significance level (alpha) of 0.05 (two-tailed), target power of 0.8, and standard effect size of 0.6. Prevalence was set to 15%. This gave us the estimation that 45 samples in each tested group is sufficient for the statistical analysis. P < 0.05 was considered statistically significant.

## Results

Gram-positive and Gram-negative groups included 50 samples, while the control group included 20 samples. The average age of patients in the Gram-positive group was 63 (M = 62.9±10.9), and in the Gram-negative group, the average age was 62 (M = 62.3 ± 17.1). There were 28 (56%) female and 22 (44%) male patients in the Gram-positive group. In the Gram-negative group, there were 30 (60%) female and 20 (40%) male patients. The control group consisted of five (25%) women and 15 (75%) men, while the average age was slightly lower compared to the Gram-positive and Gram-negative groups.

When analyzing the inflammatory parameters, the highest values of PCT, CRP, and neutrophil leukocytes were found in the Gram-negative group of bacteria (p < 0.01). PCT and CRP were also significantly higher in the Gram-negative group of samples compared to the Gram-positive group of bacteria (p < 0.001). No significant differences (p = 0.100) in CRP values were found between the Gram-positive and control groups (Table 1).

**Table 1:** Values of inflammatory parameters in the Gram-positive G (-), Gram-negative G (+), and control groups *p < 0.05 between the G (-) and G (+) groups; **p < 0.05 between the G (-) and G (+) groups and between the G (+) and control groups (ANOVA) Abbreviations: PCT: procalcitonin, CRP: C-reactive protein, G (-): Gram-negative hemoculture, G (+): Gram-positive hemoculture, M: arithmetic mean, SD: standard deviation, df: degrees of freedom, F: ratio, p: significance level

	G (-) (n = 50)	G (+) (n = 50)	Control group (n = 20)			
	M ± SD	M ± SD	M ± SD	df	F	p
PCT (ng/mL)	29.55±45.101	3.84±5.402	0.099±0.892	2	12.224	<0.001**
CRP (mg/L)	204.99±97.43	126.04±78.62	116.63±60.17	2	13.679	<0.001*
Number of leukocytes	14.76±13.95	13.56±6.17	10.56±4.26	2	1.701	0.187
Number of neutrophil leukocytes	12.02±7.40	9.76±4.94	8.28±3.88	2	4.669	0.011*
Neutrophil leukocytes (%)	80.59±15.27	79.01±16.32	74.75±12.04	2	1.049	0.354

The number of thrombocytes (PLT) was lowest in the Gram-negative group of samples. Significant differences were found between the Gram-negative group of bacteria and the control group (p = 0.008) and between the Gram-positive group and the control group (p = 0.002). Plateletcrit (Pct) was also significantly lower in the Gram-negative group compared to the Gram-positive group (p = 0.004) and compared to the control group (p = 0.022). No significant differences in PLT and Pct were found between the Gram-negative and Gram-positive groups of bacteria (p = 1.00). Significant differences in MPV were found between the Gram-negative and Gram-positive groups of bacteria, with higher MPV values ​​in the Gram-negative group (p ≤ 0.001). Significant differences in MPV were found between the Gram-negative and control groups (p = 0.047), while no significant difference in MPV was found between the Gram-positive and control groups (p = 1.000). No differences were found in MPV between the Gram-negative and Gram-positive groups (p = 0.738) (Table 2).

**Table 2:** Values of platelet indices in the Gram-positive G (-), Gram-negative G (+), and control groups Shown are means ± standard deviations, *p < 0.05; **p < 0.01 (ANOVA). Abbreviations: M: arithmetic mean, SD: standard deviation, df: degrees of freedom, F: ratio, P: significance level, PLT: number of thrombocytes, Pct: plateletcrit, MPV: mean platelet volume, PDW: platelet distribution width, P-LCR: platelet-large cell ratio, fL: femtoliter

	G (-) (n = 50)	G (+) (n = 50)	Control group (n = 20)	Df	F	p
	M ±SD	M ±SD	M ±SD			
PLT (10^9/L)	186.04±90.36	196.08±104.63	277.05±109.31	2	6.323	0.002**
Pct (%)	0.177±0.069	0.218±0.137	0.289±0.085	2	8.160	<0.001**
MPV (fL)	11.21±1.14	10.19±1.58	10.36±0.86	2	8.142	<0.001**
PDW (fL)	15.7±2.65	16.48±3.81	12.2±1.80	2	13.843	<0.001**
P-LCR (%)	36.38±7.75	30.90±9.29	30.41±6.58	2	6.767	0.002**

The PDW was significantly higher in the Gram-negative and Gram-positive groups compared to the control group (p ≤ 0.001). P-LCR values ​​were significantly higher in the Gram-negative group compared to the Gram-positive group (p = 0.004) and compared to the control group (p = 0.022). No significant differences (p = 0.100) in P-LCR were found between the Gram-positive and control groups (Table 3).

**Table 3:** Inflammatory parameters and thrombocyte indices in the Gram-negative group of bacteria *p < 0.05; **p < 0.01 (ANOVA) Abbreviations: M: arithmetic mean, SD: standard deviation, df: degrees of freedom, F: ratio, p: significance level, Lkc: leukocytes, CRP: C-reactive protein, Pct: plateletcrit, PLT: platelets, PCT: procalcitonin, MPV: mean platelet volume, PDW: platelet distribution width, P-LCR: platelet-large cell ratio, fL: femtoliter

	Escherichia coli	Klebsiella pneumoniae	Acinetobacter baumannii			
	M ± SD	M ± SD	M ± SD	df	F	p
Lkc (10^9/L)	10.37±8.48	11.59±5.10	13.53±8.16	2	0.785	0 .462
Neutrophil granulocytes (10^9/L)	8.63±8.40	22.12±8.84	13.08±8.15	2	4.214	0.021
Neutrophil granulocytes (%)	77.36±19.32	86±5.42	78.52±16.91	2	1.534	0.226
PLT (10^9/L)	172.86±86.87	203.75±112.09	181.1±93.46	2	0.476	0.624
PDW (fL)	16.04±2.95	14.14±2.56	16.64±2.14	2	3.569	0 .036
MPV (fL)	10.85±0.9	11.65±1.14	11.12±1.22	2	2.027	0 .143
P-LCR (%)	34.67±6.51	37.46±6.51	36.70±8.44	2	0.506	0.606
Pct (%)	0.18±0.06	0.17±0.03	0.18±0.02	2	0.162	0.851
CRP (mg/L)	207.55±88.38	190.41±104.63	214.87±101.08	2	0.276	0.758
PCT (ng(mL)	22.53±27.56	33.056±41.41	31.53±59.23	2	0 .234	0.792

The highest values of platelets (PLT) were found in sepsis caused by *Klebsiella pneumoniae* (M = 203.75), then in *Acinetobacter* *baumannii *(M = 181.10) and the lowest in *Escherichia coli* (M = 172.86). The PDW value was the highest in *Acinetobacter baumannii *(M = 16.64) (fL), then in *Escherichia coli *(M = 16.03) and lowest in *Klebsiella pneumoniae* (M = 14.14.) MPV (fL) and P-LCR were highest in *Klebsiella* *pneumoniae *(M = 11.65; M = 37.46), then in *Acinetobacter baumannii *(M = 11.12; M = 36.72) and lowest in *Escherichia coli* (M = 10.85; M = 34.67). Plateletcrit (Pct) was M = 0.16 in *Klebsiella pneumoniae* sepsis and was M = 0.18 in *Acinetobacter baumannii *and* Klebsiella pneumoniae *sepsis (Table 4).

**Table 4:** Inflammatory parameters and thrombocyte indices in the Gram-positive group of bacteria *p < 0.05; **p < 0.01 (ANOVA) Abbreviations: M: arithmetic mean, SD: standard deviation, df: degrees of freedom, F: ratio, P: significance level, Lkc: leukocytes, CRP: C-reactive protein, Pct: plateletcrit, CRP: C-reactive protein, number of leukocytes, neutrophil leukocytes, neutrophil leukocytes (%), PLT: platelets, PCT: procalcitonin, MPV: mean platelet volume, PDW: platelet distribution width, P-LCR: platelet-large cell ratio

	Streptococcus pyogenes	*Enterococcus* spp.	Staphylococcus aureus			
	M ± SD	M ± SD	M ± SD	df	F	p
Lkc (10^9/L)	12.87±5.83	13.64±8.63	13.57±6.17	2	0.042	0.959
Neutrophil granulocytes (10^9/L)	11.98±5.50	7.47±4.14	10.97±4.88	2	3.775	0.030*
Neutrophil granulocytes (%)	83.83±9.663	73.60±19.66	81.96±14.02	2	1.765	0.182
PLT (10^9/L)	228.17±107.61	198.94±86.38	196.08±104.64	2	0.384	0.683
PDW (fL)	15.36±2.37	15.78±2.70	17.29±4.66	2	1.141	0.328
MPV (fL)	10.12±1.95	10.28±1.73	10.14±1.43	2	0 .048	0.953
P-LCR (%)	29.48±7.72	29.99±10.08	31.93±9.26	2	0.304	0.740
Pct (%)	0.22±0.10	0.23±0.16	0.21±0.13	2	0.039	0.962
CRP (mg/L)	136.67±34.69	118.64±77.71	129.11±88.08	2	0.153	0.859
PCT (ng/mL)	1.01±0.58	3.07±0.42	5.15±0.64	2	1.848	0.169

Significant differences were found in the number of neutrophil granulocytes (#) between the three groups of positive bacteria. Between *Streptococcus pyogenes *and *Enterococcus spp.*, significantly higher values were found in the number of neutrophil granulocytes (#) in *Streptococcus pyogenes *(p = 0.030). No statistically significant differences were found in the other parameters.

When analyzing correlation, it was found that patients with *Escherichia coli *bacteria had a statistically significant positive correlation between leukocytes and P-LCR (r = 0.589; p < 0.05), neutrophil granulocytes and P-LCR (r = 0.598; p < 0.05), and neutrophil granulocytes expressed in % with P-LCR (r = 0.649; p < 0.05). By increasing the number of Lkc, neutrophil granulocytes, and neutrophil granulocytes expressed in %, P-LCR also increases in the *Escherichia coli *group (Table 5).

**Table 5:** Correlation between platelet indices and leukocyte count, proportion of neutrophil granulocytes, procalcitonin (PCT), and C-reactive protein (CRP) in sepsis caused by Gram-negative G (-) bacteria (Escherichia coli, Klebsiella pneumoniae, and Acinetobacter baumannii) Abbreviations: Lkc: leukocytes, fL: femtoliter *p < 0.05; **p < 0.01 (Spearman correlation coefficient)

Group of the bacteria		PLT	PDW	MPV	P-LCR	Pct
Escherichia coli	Lkc (10^9/L)	0.440	0.452	0.104	0.589*	0.170
	Neutrophil granulocytes (10^9/L)	0.520	0.416	0.087	0.598*	0.092
	Neutrophil granulocytes (%)	0.183	0.385	0.260	0.649*	0.168
	PCT (ng/mL)	0.042	-0.074	0.182	0.033	-0.269
	CRP (mg/L)	-0.209	0.389	0.528	0.136	-0.086
Klebsiella pneumoniae	Lkc (10^9/L)	-0.228	-0.099	0.646**	0.400	-0.550 *
	Neutrophil granulocytes (10^9/L)	0.229	0.116	-0.037	-0.031	-0.121
	Neutrophil granulocytes (%)	-0.109	0.021	0.308	0.176	-0.129
	PCT (ng/mL)	-0.351	0.203	0.228	0.203	0.039
	CRP (mg/L)	-0.526 *	0.443	0.137	0.348	0.052
Acinetobacter baumannii	Lkc (10^9/L)	0.097	0.381	-0.398	-0.229	-0.305
	Neutrophil granulocytes (10^9/L)	0.198	0.245	-0.476	-0.377	0.050
	Neutrophil granulocytes (%)	0.226	0.165	-0.231	-0.079	-0.081
	PCT (ng/mL)	-0.090	-0.041	0.187	-0.033	-0.057
	CRP (mg/L)	0.059	-0.193	-0.066	-0.009	0.238

A statistically significant positive correlation was found between leukocytes and MPV (r = 0.646; p < 0.01) in patients with *Klebsiella* *pneumoniae *bacterial infection where an increased number of leukocytes, correlated with an increase in MPV. A statistically significant negative correlation was found between CRP and PLT (r = -0.526; p < 0.05), where a decrease in CRP is correlated with an increase in PLT. Patients with *Acinetobacter baumannii *bacteria had a statistically significant negative correlation between neutrophil granulocytes and MPV (r = -0.476; p < 0.05). A statistically significant negative correlation was found in samples with *Klebsiella* *pneumonia*e between plateletcrit (PCT) and Lkc (r = -0.550; p < 0.05) (Table 6).

**Table 6:** Correlation between platelet indices and leukocyte count, proportion of neutrophil granulocytes, procalcitonin (PCT), and C-reactive protein (CRP) in sepsis caused by Gram-positive bacteria G (+) (Streptococcus pyogenes, Enterococcus spp., and Staphylococcus aureus) Abbreviations: Lkc: leukocytes, fL: femtoliter *p < 0.05; **p < 0.01 (Spearman correlation coefficient)

Group of the bacteria		PLT	PDW	MPV	P-LCR	Pct
Streptococcus pyogenes	Lkc (10^9/L)	0.496	-0.620	0.114	-0.081	0. 582
	Neutrophil granulocytes (10^9/L)	0.338	-0.496	-0.111	-0.365	-0.342
	Neutrophil granulocytes (%)	-0.596	0.239	-0.029	-0.467	-0.550
	PCT (ng/mL)	0. 326	-0.305	0.346	0.374	0.809
	CRP (mg/L)	0.615	-0.530	-0.589	-0.505	0.466
Enterococcus spp.	Lkc (10^9/L)	-0.172	0.376	-0.165	-0.271	0.515^*^
	Neutrophil granulocytes (10^9/L)	0.498^*^	0.081	0.193	0.128	-0.485^*^
	Neutrophil granulocytes (%)	0.493^*^	-0.275	0.139	0.267	-0.245
	PCT (ng/mL)	0.466^*^	0.624^**^	0.164	-0.087	0.024
	CRP (mg/L)	0.002	0.049	0.123	0.204	0.130
Staphylococcus aureus	Lkc (10^9/L)	-0.071	0.000	0.163	0.190	0.817^**^
	Neutrophil granulocytes (10^9/L)	-0.135	0.154	0.351	0.445^*^	-0.391
	Neutrophil granulocytes (%)	-0.209	0.217	0.356	0.508^**^	-0.107
	PCT (ng/mL)	-0.137	-0.077	0.101	-0.328	-0.327
	CRP (mg/L)	-0.440^*^	0.086	-0.131	0.260	0.277

A statistically significant positive correlation was found between PCT and PDW (r = 0.624; p < 0.01) in patients with *Enterococcus spp* bacterial infection where an increased number of leukocytes is correlated with an increase in MPV. A statistically significant negative correlation was found between neutrophil granulocytes (#) and PCT, neutrophil granulocytes (%), and PLT (r = 0.498; r = 0.493; r = 0.466; p < 0.05), where a decrease in neutrophil granulocytes (#) is correlated with an increase in Pct. Patients with *Staphylococcus aureus *bacteria had a statistically significant negative correlation between CRP and PLT (r = -0.440; p < 0.05).

To determine the diagnostic possibility of predicting sepsis, the receiver operating characteristic (ROC) analysis was used (Table 7). Furthermore, ROC curve analysis was employed to ascertain the diagnostic efficacy of combined markers detection in sepsis. A comparison of combined parameters detection was conducted using a z-test, with statistical significance defined as p < 0.05 (Table 7).

**Table 7:** Results of the ROC analysis for different markers in the prediction of sepsis compared to the control group Abbreviations: PLT: platelets, MPV: mean platelet volume, P-LCR: platelet-large cell ratio, PDW: platelet distribution width, fL: femtoliter *p < 0.05; **p < 0.01 Source: edited by the author

Variables	AUC	SE ^a^	95% CI ^b^	p-value
PLT (10^9^/L)	0.750	0.0617	0.632 to 0.846	0.001*
MVP (fL)	0.743	0.0647	0.625 to 0.840	0.0002**
P-LCR (%)	0.710	0.0697	0.589 to 0.812	0.0026**
PDW (fL)	0.865	0.0443	0.762 to 0.935	0.001**

**Figure 1:**
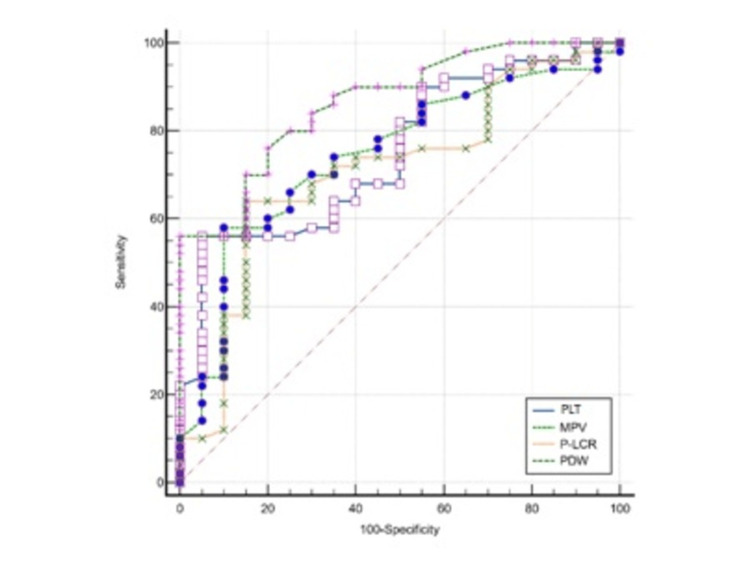
Results of the receiver operating characteristic (ROC) analysis for different markers in the prediction of sepsis compared to the control group. *p < 0.05; **p < 0.01 Abbreviations: PLT: platelets, MPV: mean platelet volume, P-LCR: platelet-large cell ratio, PDW: platelet distribution width

The ROC curve analysis was performed with the aim of testing the possibility of establishing a diagnosis of sepsis based on platelet indices, namely, PLT, PDW, MPV, and P-LCR (Figure 1).

In addition to determining the values under the ROC curves (AUC), specificity, sensitivity, positive predictive value (PPV), negative predictive value (NPV), and accuracy were also calculated for platelets, mean platelet volume, and platelet distribution.

The area under the curve for the diagnosis of sepsis of PLT, MVP, P-LCR, and PDW was 0.750, 0.743, 0.710, and 0.865, respectively. This result implies that the diagnostic efficacy of PDW was better than PLT, MVP, and P-LCR. The sensitivity and specificity of PLT, MVP, P-LCR, and PDW in the group of Gram-negative bacteria were 85.11%, 74.47%, 71.11%, and 66.67%, respectively.

## Discussion

The main findings of our study are that patients with sepsis have decreased numbers of thrombocytes and plateletcrit and increased values of MPV, PDW, and P-LCR, indicating an increase in thrombocyte production. Moreover, the results were more prominent in sepsis caused by Gram-negative bacteria, i.e., *E. coli*, *K. pneumoniae*, and *A. baumannii*, compared to sepsis caused by Gram-positive bacteria, i.e., *S. pneumoniae*, *Enterococcus spp*., and methicillin-resistant *Staphylococcus aureus* (MRSA). When comparing Gram-negative negative bacteria, the platelet count (PLT) was lowest in sepsis caused by *E. coli*, followed by *A. baumannii* and *K. pneumoniae*. The PDW value was highest in *A. baumannii*, followed by *E. coli*, and lowest in *K. pneumoniae*. MPV and P-LCR were highest in *K. pneumoniae*, followed by *A. baumannii*, and lowest in *E. coli*. The values of procalcitonin were significantly higher in patients with Gram-negative sepsis, meaning stronger activation of inflammatory response in those patients. The total number of leukocytes and the number of neutrophile leukocytes were not different among groups. In addition, the highest values of procalcitonin were in sepsis caused by *K. pneumoniae* and CRP in sepsis caused by *A. baumannii*.

Sepsis and septic shock result from inflammatory responses to microbial components, involving cytokine cascades, leukocyte-induced damage, endothelial injury, peripheral vasodilation, and disseminated intravascular coagulation [1]. Our results are in line with other studies, which showed no differences in the number of leukocytes and neutrophil leukocytes among three tested groups of patients with sepsis [3,22,23,27]. While significantly higher levels of procalcitonin and CRP were recorded in sepsis caused by Gram-negative and Gram-positive bacteria when compared to the control group [27]. There is a set of markers that could be considered as a predictor of sepsis mortality. The degree of thrombocytopenia is the indicator of a poor prognosis, associated with an increased risk of bleeding, greater organ dysfunction, and suboptimal sepsis treatment outcomes. In addition, other coagulation and platelet function parameters are altered in septic shock, as evidenced by prolonged activated partial thromboplastin time and prothrombin time, along with reduced platelet counts. Research indicates that approximately half of sepsis patients exhibit elevated MPV [28]. Furthermore, some studies have reported increased MPV and PDW, while PLT and Pct are reduced in sepsis patients compared to control groups [28]. Conversely, other studies have observed a decrease in MPV and PLT in sepsis. Nonetheless, the precise impact of Gram-negative bacterial sepsis on platelet indices remains insufficiently understood. The number of platelets increases due to inflammatory cytokines, which are elevated during sepsis, while their degradation mainly occurs in the spleen, an essential immune organ. Consequently, various inflammatory mediators in sepsis can influence platelet count and the indices related to platelet production and degradation [15-18]. 

Different mechanisms of thrombocytopenia in sepsis are proposed: decreased production of thrombocytes, platelet sequestration, consumptive coagulopathy, and hemodilution [15]. One of the first proposed mechanisms was a decrease in thrombocyte production due to the toxic effect of sepsis on the bone marrow [16]. Meanwhile, another research proposed an increased production of thrombocytes in sepsis, as evidenced by increased values of thrombopoietin in septic patients [17]. In addition, reticulated platelet percentage and thrombopoietin were increased in septic patients, meaning an increase in platelet production rate [18]. In other studies, it has been proposed that different pro-inflammatory markers such as tumor necrosis factor-α (TNF-α), interleukin (IL)-1, IL-6, and IL-8 are increased in sepsis and may lead to an increase in thrombocyte production [19]. Different bacteria, such *Streptococcus sanguis*, *Staphylococcus aureus*, and *Borrelia burgdorferi*, may bind platelets via fibrinogen, glycoprotein IIb/IIIa (GPIIb/IIIa), and GPIb thrombocyte receptors, leading to an increase in thrombocyte aggregation [19-21]. This mechanism could be one of the causes of thrombocytopenia in septic patients. Other mechanisms of thrombocytopenia in sepsis such as consumptive coagulopathy and platelet sequestration are less likely to be direct effects of bacteria on thrombocytes [22]. Different medications are used in sepsis patients, such as antiplatelet agents (e.g., acetylsalicylic acid, clopidogrel) or anticoagulants (e.g., heparin). These medications may influence platelet count and function, potentially affecting the platelet parameters that we investigated. In addition, patients with prolonged sepsis may experience impaired bone marrow function, which could adversely impact platelet production. Earlier studies have shown that the increased value of PDW is associated with increased mortality in patients with severe sepsis [25]. Also, MPV is increased in the early days of sepsis caused by Gram-positive and Gram-negative sepsis [25]. A study with a larger cohort that examines infection outcomes, various therapeutic modalities, and specific pathogens could further enhance the understanding of platelet dysfunction in sepsis.

Our results demonstrated a statistically significant positive correlation between leukocytes and MPV in patients with *K. pneumoniae* infections, where an increased leukocyte count corresponded with an elevated MPV. In addition, a statistically significant negative correlation was observed between CRP and PLT, indicating that a decrease in CRP was associated with an increase in PLT. In patients with *A. baumannii* infections, a statistically significant negative correlation was found between neutrophil granulocytes and MPV [22,23,25].

Based on the calculated correlation coefficients, it can be concluded that there is a significant correlation between the determined parameters, markers of sepsis, with individual platelet indices. ROC analysis strongly suggests that platelet indices may be considered a representative prognostic indication for sepsis. Disturbances in the value of these markers may reflect the infectious state and the degree of intoxication [18,25]. Testing the relationship between platelet indices with the values of procalcitonin, CRP, leukocyte count, and the proportion of neutrophil leukocytes in sepsis caused *by E. coli* indicates a positive statistically significant correlation between leukocytes and P-LCR and neutrophil granulocytes and P-LCR. Patients with *K. pneumoniae* bacteria have a statistically significant positive correlation between leukocytes and MPV. Statistically significant negative correlations between neutrophil gram PDW are the highest in *A. baumannii*, then in *E. coli*, and lowest in *K. pneumoniae*. Values of MPV and P-LCR are highest in *K. pneumoniae*, then in *Acinetobacter baumannii*, and lowest in *E. coli*. ROC analysis revealed that platelet indices can serve as prognostic indicators for sepsis.

The limitation of our study it is a single-center study and a relatively small number of samples, samples from three specific clinical departments with high numbers of infections. Thus, the results should be investigated in multicentric studies with a higher number of patients coming from different clinical departments and including other bacteria as a cause of sepsis.

## Conclusions

The results from our study show that patients with sepsis have a decreased PLT and Pct and increased values of MPV, PDW, and P-LCR, indicating an increase in thrombocyte production. In addition, the results were more prominent in sepsis caused by Gram-negative bacteria compared to sepsis caused by Gram-positive bacteria. Therefore, platelet indices may be potentially useful diagnostic or prognostic markers in bacterial sepsis, yet their use can have certain limitations due to their change in different pathological conditions. Future studies are needed to explore in more detail the underlying mechanism that may lead to the altered function of thrombocytes in patients with sepsis. This could help in developing different drugs that may improve the function of thrombocytes in septic patients.
